# Attraction-Mediated Synergy: Insecticide Toxicity Against *Coptotermes formosanus* Enhanced by *Trichoderma* Metabolites

**DOI:** 10.3390/insects16111116

**Published:** 2025-10-31

**Authors:** Aysha Siddika, Siqi Chen, Keer Zhu, Xiangfei Wang, Xinquan Du, Linjuan Wan, Min Liu, Lang Zhang

**Affiliations:** School of Pharmacy/Key Laboratory of Xinjiang Phytomedicine Resource and Utilization, Ministry of Education/Institute for Safflower Industry Research, Shihezi University, Shihezi 832003, China; asnourin200@gmail.com (A.S.); sq153181@163.com (S.C.); zhukeer386@gmail.com (K.Z.); wxf_601@shzu.edu.cn (X.W.); duxinquan2025@163.com (X.D.); wlj1106@shzu.edu.cn (L.W.)

**Keywords:** *Coptotermes formosanus*, *Trichoderma* metabolites, attraction behavior, insecticides, synergistic

## Abstract

**Simple Summary:**

Previous research showed that *Trichoderma* metabolites trigger aggregation behavior in the Formosan subterranean termite, *Coptotermes formosanus*. This study demonstrates that three specific metabolites—phenol, ethyl 2,4-dioxovalerate, and diglycolic acid—act as effective attractants and behavioral regulators for termites without exhibiting significant intrinsic toxicity. These metabolites stimulated trail-following and aggregation behaviors in termites. When combined with imidacloprid, they significantly enhanced termite mortality compared to insecticide treatments alone. Notably, these metabolites also attracted termites to areas treated with insecticides (imidacloprid or fipronil), resulting in elevated mortality and demonstrating a strong “attract-and-kill” effect. The findings indicate that *Trichoderma* metabolites are promising and environmentally safe candidates for improving termite control strategies by synergistically enhancing insecticidal efficacy through behavioral manipulation.

**Abstract:**

Previous studies have demonstrated that *Trichoderma* metabolites triggered aggregation behavior in *Coptotermes formosanus* (Blattodea: Rhinotermitidae). Building on this, the present work systematically evaluated the behavioral effects of three specific *Trichoderma* metabolites—phenol, ethyl 2,4-dioxovalerate, and diglycolic acid—and their synergistic interactions with insecticides. We hypothesized that these metabolites attract *C. formosanus* through multiple behavioral mechanisms and enhance the toxicity of insecticides. Bioactivity showed that ethyl 2,4-dioxovalerate and diglycolic acid exhibited no significant toxicity. Phenol (5 × 10^−2^ to 5 µg/cm) and ethyl 2,4-dioxovalerate (5 × 10^−1^ µg/cm) elicited trail-following behavior. In the no-choice insecticide synergy test, phenol or diglycolic acid combined with imidacloprid (50 µg/g) resulted in substantially higher mortality compared to insecticides alone. The combination of metabolites with fipronil resulted in 100% mortality in termites. In two-choice aggregation tests, termite presence on metabolite-treated filter papers was significantly elevated compared to the controls. Fipronil (10 μg/g) alone significantly reduced termite aggregation. But when fipronil was combined with the metabolites, termite presence on the treated papers increased significantly, resulting in a substantial rise in mortality and demonstrating a clear attract–kill synergy. These findings identify *Trichoderma* metabolites as safe and effective behavioral regulators for *C. formosanus*. By enhancing insecticidal efficacy through attractant–toxicity synergy, they represent promising candidates for developing novel termite control strategies.

## 1. Introduction

Formosan subterranean termites (Blattodea: Rhinotermitidae) cause annual global economic losses amounting to USD 40 billion, with China alone accounting for USD 1 billion [1,2]. Within the family Rhinotermitidae, species of the genus *Coptotermes* are particularly problematic, contributing to approximately 40% of total global termite-related losses [3]. *Coptotermes formosanus* Shiraki stands out as one of the most economically destructive insect species worldwide [2,4]. This species, predominantly found in tropical and subtropical regions globally, voraciously consume cellulose-based materials such as wood, progressively degrading residential buildings, eroding cultural heritage sites, disrupting agricultural and forestry ecosystems, and compromising vital water supply networks and electrical installations [5,6,7,8,9]. Due to the large number of colony populations (exceeding millions), expansive foraging territories (up to 3571 m^2^), rapid reproduction rates, and hidden nests, it is difficult to control termites in time before they are infested on the ground [10,11,12,13].

Currently, liquid termiticides and bait remains one of the main methods for controlling subterranean termites [9]. Liquid termiticides establish soil barriers through repellent or poison termites to prevent termite invasion, predominantly employed for spatially targeted protection; bait combines slow-acting insecticides with attractants, enabling colony-wide toxicant dissemination via trophallaxis among termites, making them suitable for large-scale termite management [3,14,15,16]. These two methods can complement each other. However, the soil barrier is constrained by termite–insecticide contact, while bait is restricted by the lack of highly active termite attractants. To enhance termite control efficacy, numerous studies have been conducted to identify potent attractants that elicit termite behavioral responses such as aggregation, trail following, feeding, and tunneling behaviors, which could be helpful for termites to locate baits or increase insecticide exposure and improve the effectiveness of termite control. For example, 2-phenoxyethanol, a chemical component of ballpoint pen ink, could elicit trail-following and tunneling behaviors in *C. formosanus* when synergistic application with termiticides (acetamiprid, fipronil, and imidacloprid) significantly enhanced termite–insecticide contact frequency and mortality [17,18,19].

Our previous metabolites profiling of *Trichoderma* species identified multiple aggregation-inducing metabolites targeting *C. formosanus* [20]. Throughout the experimental period, phenol (1000 μg/mL) and diglycolic acid (1000 μg/mL) produced by *Trichoderma asperellum*, as well as ethyl 2,4-dioxovalerate (1000 μg/mL) —specifically produced by *Trichoderma virens—*were observed to consistently elicit the aggregation behavior of *C. formosanus* [20]. Furthermore, ethyl 2,4-dioxovalerate was also found to elicit tunneling behavior in termites, while combinatorial application with fipronil exhibited synergistic potentiation of its toxic efficacy [21].

These *Trichoderma* metabolites could enhance termite bait detection or increase insecticide exposure, thereby improving termite control efficacy. This study demonstrates that phenol, ethyl 2,4-dioxovalerate, and diglycolic acid may attract *C. formosanus* in various ways and can be used for termite prevention and control. We hypothesized that (1) phenol, ethyl 2,4-dioxovalerate, and diglycolic acid may attract *C. formosanus* through aggregation, trail-following, and feeding behaviors in termites without compromising physiological vitality (survivorship) and (2) phenol, ethyl 2,4-dioxovalerate, and diglycolic acid may potentiate insecticidal activity through synergistic attractant-toxicity effects. To validate these hypotheses, we first conducted the no-choice test, two choice trail-following test, and three-choice feeding preference test to investigate the effects of these three compounds on the physiological activity and attractive behaviors of *C. formosanus*. No-choice insecticide synergy tests were implemented to assess the synergistic effects of these three compounds on insecticides. In addition, two-choice aggregation tests were performed to evaluate the attract–kill effects of the combination of these three compounds and insecticides on *C. formosanus*.

## 2. Materials and Methods

### 2.1. Termites

Three colonies of *C. formosanus* (>1 km apart from each other) were collected in the arboretum of South China Agricultural University (Guangzhou, China) [22]. The termites were stored in rectangular plastic containers (55 cm long, 40 cm wide, 31 cm high) with pine wood blocks at room temperature (25 ± 2 °C). Termite colonies comprised workers and soldiers at a ratio of 47:3 and were used within 1 month of collection.

### 2.2. Preparation of Pine Wood Blocks and Filter Paper

Three *Trichoderma* metabolite standard compounds, including phenol (99%), ethyl 2, 4-dioxovalerate (97%), and diglycolic acid (98%), were purchased from Shanghai Macklin Biochemical Co., Ltd. (Shanghai, China) and dissolved in analytical-grade acetone (Fuyu Chemical Co., Ltd., Tianjin, China). In the no-choice test, solutions of metabolites were prepared in acetone at concentrations of 0.75, 7.5, 75, 750, and 7500 μg/mL, and 200 μL of solution was applied dropwise onto rectangular pine wood blocks (2 × 2 × 0.3 cm; average weight 0.6 g). In the three-choice feeding preference test, metabolites were prepared in acetone at concentrations of 0.16, and 1.6 mg/mL, and 500 μL of solution was applied dropwise onto pine wood blocks (square length: 2.0 cm; average weight: 3.2 g). After the acetone completely evaporated for 2 h, final metabolite concentrations of 0.25, 2.5, 25, 250, and 2500 μg/g were observed on the treated wood blocks. Pine wood slices treated with pure acetone (no metabolites) were used as a control (0 μg/g).

Imidacloprid (97%) was purchased from Shanghai Macklin Biochemical Co., Ltd. (Shanghai, China), and fipronil (25 g/L) was purchased from Bayer Crop Science Inc. (Beijing, China). Based on reports that fipronil exhibited a lower median lethal concentration (LC_50_) against termites compared to imidacloprid [23], these two insecticides were separately prepared as acetone solutions at various concentrations. The concentrations of imidacloprid were 11.15 and 22.30 μg/mL, while the concentrations of fipronil were 0.446 and 4.46 μg/mL. Metabolites were also dissolved separately in acetone at a concentration of 1115 μg/mL. In the no-choice insecticide synergy test, about 1 mL of solution was applied to filter paper (diameter: 8.5 cm; weight: 0.4460 g) and allowed to dry for 30 min until the acetone had completely evaporated. In the two-choice aggregation test, about 100 μL of solution was applied to filter paper (diameter: 2.5 cm; weight: 0.0446 g) and allowed to dry for 10 min until the acetone had completely evaporated. Each solution—containing the metabolite alone, insecticide alone, or a mixture of both—was applied uniformly to the filter paper. The following treated filter papers were prepared: metabolite-only (2500 μg/g), imidacloprid-only (25 or 50 μg/g), fipronil-only (1 or 10 μg/g), metabolite (2500 μg/g) combined with imidacloprid (25 or 50 μg/g), and metabolite (2500 μg/g) combined with fipronil (1 or 10 μg/g). A control group (0 μg/g) was prepared using filter paper treated with acetone only.

### 2.3. Bioactivity of Trichoderma Metabolites Against Coptotermes formosanus

#### 2.3.1. Experiment 1: Effects of *Trichoderma* Metabolites on Survivorship of *Coptotermes formosanus* (No-Choice Test)

This study conducted a no-choice test to evaluate the survivorship, body water content, and wood consumption of *C. formosanus* after feeding on pine wood blocks. Each metabolite was tested at five concentrations (0.25, 2.5, 25, 250, and 2500 μg/g), with untreated pine wood blocks (0 μg/g) serving as the control. The experimental arena consisted of a Petri dish (9.0 cm in diameter) containing a centrally placed rectangular pine wood block. Fine sand was sifted through a 0.85 mm sieve, washed, baked at 80 °C for 3 d, and dried at 50 °C for one week. The pine wood blocks were baked at 50 °C for one week prior to use. Each Petri dish was filled with 30 g of sand (15% water content, calculated as [water weight/dry soil weight] × 100) with a wood block. A total of 50 termites (47 workers and 3 soldiers) were released into each dish and incubated at 25 ± 2 °C. Each treatment was replicated 15 times (5 replicates × 3 colonies). After 14 d, the number of live termites were counted.

#### 2.3.2. Experiment 2: Effects of *Trichoderma* Metabolites on Trail-Following Behavior in *Coptotermes formosanus* (Two-Choice Trail-Following Test)

This study investigated the effect of *Trichoderma* metabolites on trail-following behavior in termite using a two-choice test adapted from Sillam-Dussès et al. [24]. Trail-following distances were measured under different metabolite concentrations. Each metabolite was tested on a Y-shaped line (3 cm in stem, 7 cm in branch) with a 120° angle between each branch on filter paper (15 cm in diameter). Each metabolite (phenol, ethyl 2, 4-dioxovalerate, and diglycolic acid) was dissolved in acetone to prepare solutions at specified concentrations. Firstly, 10 μL of acetone was extracted using a flat-mouth micro-injector, and the path was drawn on the Y-shaped line as a control branch. After a 30 s period to allow complete acetone evaporation, the opposite branch was treated with 10 μL of the metabolite–acetone solution. The final concentrations of the metabolite on the stem and branch were 5 × 10^−4^, 5 × 10^−3^, 5 × 10^−2^, 5 × 10^−1^, 5, and 5 × 10^1^ μg/cm. The branches of the trail were randomized across replicates to avoid bias. One worker termite was placed in the release chamber (2.5 cm Petri dish with a 2 mm opening set on the end of the stem), and the distance that the termites walked along the trail was recorded (maximum distance = 10.0 cm). Each metabolite was tested 60 times (20 replicates × 3 colonies). Both the filter paper and termite were replaced after each test. A metabolite was determined to elicit trail-following activity if the mean trail-following distance exceeded 3.0 cm.

#### 2.3.3. Experiment 3: Effects of *Trichoderma* Metabolites on Feeding Preference in *Coptotermes formosanus* (Three-Choice Feeding Preference Test)

This study investigated the effect of *Trichoderma* metabolites on the feeding preference of *C. formosanus* using a three-choice feeding preference test. The test compared termite wood consumption between metabolite-treated square pine wood blocks and untreated control blocks (0 μg/g). The experimental arena consisted of a plastic bowl (upper diameter: 15.0 cm; lower diameter: 10.5 cm; height: 7.0 cm) containing three bait boxes (upper diameter: 4.0 cm; lower diameter: 3.0 cm; height: 3.0 cm) (Supplementary Figure S1). Each bait box was perforated with 10 holes (diameter: 5.0 mm) arranged in two staggered rows. Fine soil (72% sand, 17% silt, 11% clay) was sifted through a 2 mm sieve, baked at 80 °C for 3 days, and dried at 50 °C for one week prior to use. Treated and control wood blocks were randomly placed into bait boxes, which were buried under 350.0 g soil (17% water content) within the plastic bowl. Four hundred termites (only workers) were released into the arena and incubated at 25 ± 2 °C for 3 weeks. Each test was repeated 12 times (4 replicates × 3 colonies). Wood consumption was quantified by measuring pre/post-treatment weight differences in the pine wood blocks.

### 2.4. Synergistic Effects of Trichoderma Metabolites on Insecticidal Activity Against Coptotermes formosanus (No-Choice Insecticide Synergy Test)

We conducted a no-choice insecticide synergy test to investigate the potential of *Trichoderma* metabolites to enhance the insecticidal efficacy against *C. formosanus*. Twelve treatment groups were tested, including metabolite-only treatments, insecticide-only treatments, treatments using combinations of metabolites and insecticides, and an untreated control (filter paper without any treatment) (Table 1 and Table 2). The experimental arena comprised a Petri dish (9.0 cm in diameter) and a filter paper. Treated filter papers were placed in a Petri dish and moistened with 1 mL of sterile distilled water. A total of 50 termites (47 workers and 3 soldiers) were released into the arena and incubated at 25 ± 2 °C. Each treatment was repeated 18 times (6 replicates × 3 colonies). The number of live termites was counted for 7 consecutive days.

### 2.5. Synergistic Attract–Kill Effects of Trichoderma Metabolites Combined with Insecticides on Coptotermes formosanus (Two-Choice Aggregation Test)

This study employed the two-choice aggregation test to evaluate the effects of combined *Trichoderma* metabolites and insecticides on the aggregation preference and mortality of *C. formosanus*, aiming to investigate whether their combination enhances the attract–kill efficacy against *C. formosanus*, and the conducted using the methodology described by Zhang et al. [20]. Twelve groups were established to evaluate and compare the distribution percentages of termites on filter papers treated with metabolite-only treatments, insecticide-only treatments, combinations of metabolites and insecticides, or untreated controls, along with termite mortality within the apparatus (Table 3 and Table 4). A control group without any compound or insecticide was included, in which one filter paper was randomly designated as “treated” to serve as a blank comparison. Each treatment was repeated 18 times (6 replicates × 3 colonies). The experimental apparatus consisted of a Petri dish (9.0 cm in diameter) containing two filter papers positioned on opposite sides. Twenty-five termites (only worker) were released into the center of the dish and incubated at 25 ± 2 °C. The number of termites aggregated at different positions (treated or untreated filter paper or wandering in the Petri dish) were recorded at 1, 2, 4, 8, and 12 h. The number of live termites was counted for 7 consecutive days.

### 2.6. Statistical Analysis

In all experiments, the normality of data was assessed using the Shapiro–Wilk test. For data conforming to normality distribution (including raw data, square-root, or log transformation of raw data), a two-way ANOVA was applied with termite colony as a random effect and treatment group as a fixed effect, followed by Tukey’s HSD test for post hoc multiple comparisons. If raw data and transformed data still did not conform to the normal distribution, Kruskal–Wallis tests were performed, followed by Dunn’s test for multiple comparisons. For Experiment 2.5, the percentage data of termites at each position were subjected to log-ratio transformation to ensure data independence. The transformed data were analyzed using a two-way ANOVA (with colony as the random effect and location as the fixed effect), with Tukey’s HSD test for pairwise comparisons. For Experiment 2.4 and Experiment 2.5, probit regression was employed to estimate median lethal time (LT_50_). However, excessively high chi-square values (*χ*^2^) led to unreliable conclusions; thus, LT50 estimates were excluded from comparative analysis. All statistical procedures were analyzed using SAS 9.4 (SAS Institute, Cary, NC, USA).

## 3. Results

### 3.1. Bioactivity of Trichoderma Metabolites Against Coptotermes formosanus

#### 3.1.1. Experiment 1: Effects of *Trichoderma* Metabolites on Survivorship of *Coptotermes formosanus* (No-Choice Test)

**Figure 1:**
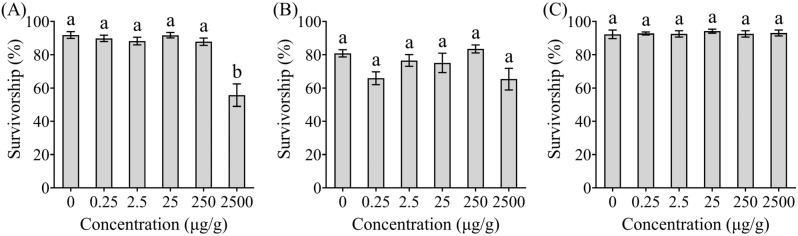
Effects of different compounds on the survivorship of *Coptotermes formosanus*. The *Trichoderma* metabolites tested in this study were phenol (**A**), ethyl 2, 4-dioxovalerate (**B**), and diglycolic acid (**C**). Data are presented as mean ± SE; different letters indicate significant differences (*p* < 0.05).

No significant differences in survivorship were observed between termites exposed to ethyl 2, 4-dioxovalerate (*χ*^2^ = 15.25; df = 5; *p* = 0.0094; Figure 1B) or diglycolic acid (*χ*^2^ = 1.65, df = 5, *p* = 0.8953; Figure 1C) compared to the control group, but termites exposed to phenol at 2500 μg/g exhibited significantly reduced survival rates (*χ*^2^ = 27.64, df = 5, *p* < 0.0001; Figure 1A).

#### 3.1.2. Experiment 2: Effects of *Trichoderma* Metabolites on Trail-Following Behavior in *Coptotermes formosanus* (Two-Choice Trail-Following Test)

**Figure 2:**
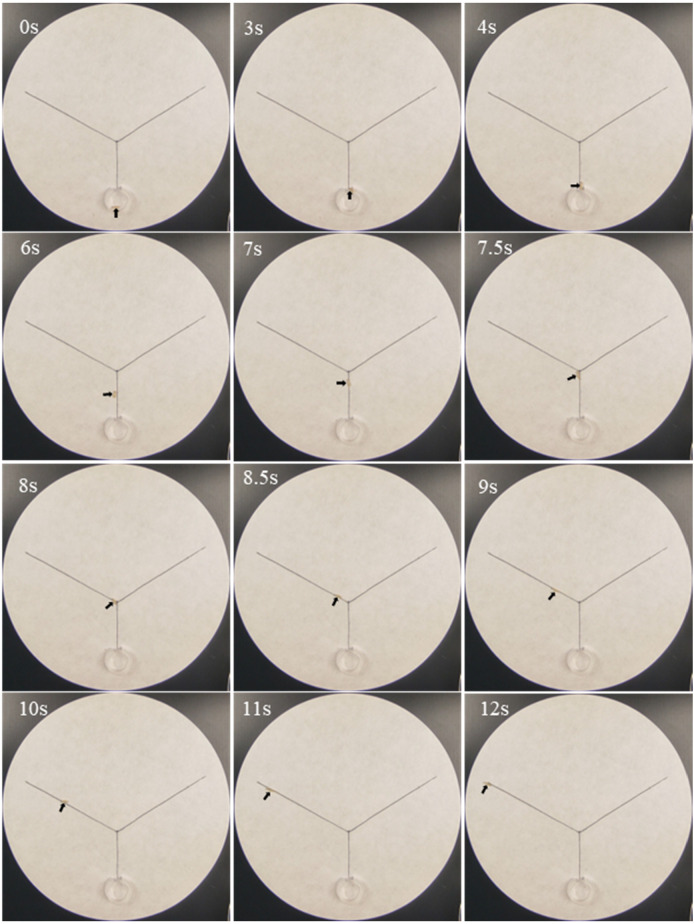
Examples of trail-following behavior of *Coptotermes formosanus* workers along paths treated with phenol (0.5 μg/cm), indicating strong trail-following activity at this concentration. In this example, the right branch was drawn with acetone as a control, whereas the left branches were drawn with phenol (the final concentration was 0.5 μg/cm). The arrows indicated the location of worker termites at different time points.

Phenol was active in eliciting trail-following behavior from 5 × 10^−2^ to 5 μg/cm (Table 5). A representative example demonstrated that at 0.5 μg/cm of phenol, termites reached the exit of the release chamber within 3 s, subsequently crawled along the stem, accessed the phenol-treated branch at 7.5 s, and reached the endpoint of the phenol trail by 12 s (Figure 2). At a concentration of 5 × 10^−1^ μg/cm, ethyl 2, 4-dioxovalerate exhibited trail-following activity, inducing a crawling distance of 3.8 ± 0.4 cm. However, no concentration of diglycolic acid (5 × 10^−4^ to 5 × 10^1^ μg/cm) enhanced trail-following activity, as evidenced by average crawling distances consistently remaining below 3.0 cm.

#### 3.1.3. Experiment 3: Effects of *Trichoderma* Metabolites on Feeding Preference in *Coptotermes formosanus* (Three-Choice Feeding Preference Test)

None of the various concentrations of the *Trichoderma* metabolites (phenol, ethyl 2, 4-dioxovalerate, and diglycolic acid) induced significant feeding preference in termites, and the wood consumption of termites on compound-treated wood blocks showed no significant difference compared to the control (Table 6).

### 3.2. Synergistic Effects of Trichoderma Metabolites on Insecticidal Control of Coptotermes formosanus (No-Choice Insecticide Synergy Test)

**Figure 3:**
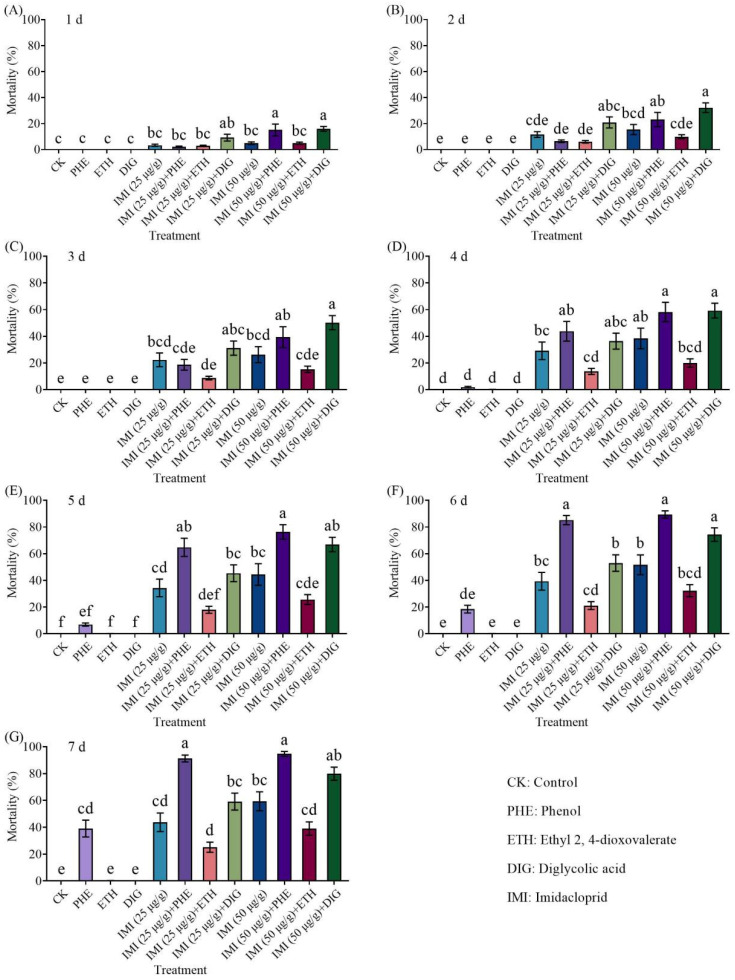
Effect of the combined application of different compounds and imidacloprid on the mortality of *Coptotermes formosanus* at 1 d (**A**), 2 d (**B**), 3 d (**C**), 4 d (**D**), 5 d (**E**), 6 d (**F**), and 7 d (**G**). Data are presented as mean ± SE; different letters indicate significant differences (*p* < 0.05).

The combined application of *Trichoderma* metabolites (phenol, ethyl 2, 4-dioxovalerate, and diglycolic acid) with imidacloprid (25 or 50 μg/g) effectively killed termites (Figure 3 and Supplementary Table S1). At 1–2 d and 7 d, the mortality of termites caused by the combination of 50 μg/g imidacloprid with diglycolic acid was significantly higher than that of imidacloprid alone. And combinations of imidacloprid (25 and 50 μg/g) with phenol resulted in significantly higher mortality compared to imidacloprid alone at 5–7 days.

**Figure 4:**
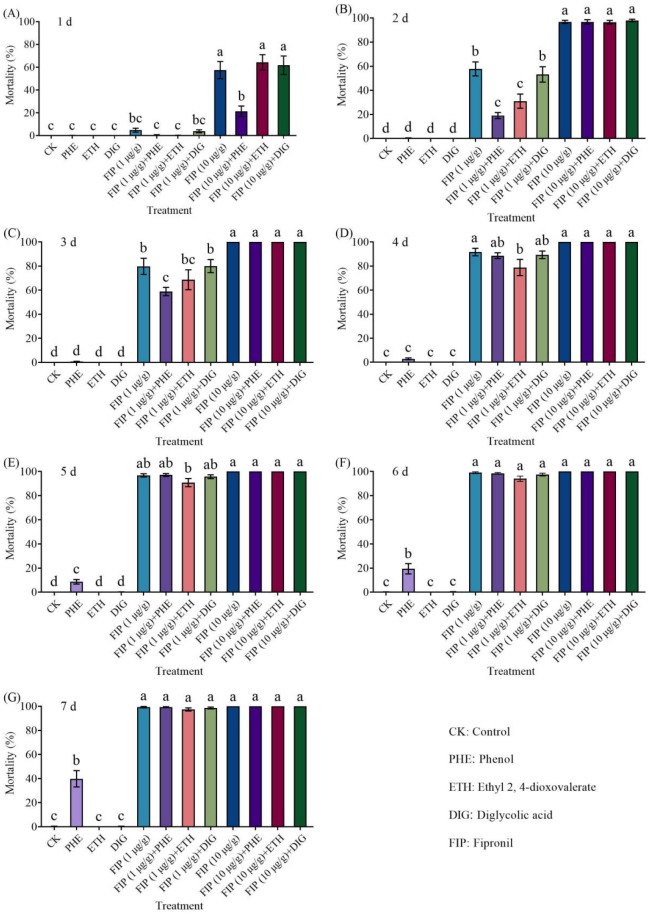
Effects of the combined application of different compounds and fipronil on the mortality of *Coptotermes formosanus* at 1 d (**A**), 2 d (**B**), 3 d (**C**), 4 d (**D**), 5 d (**E**), 6 d (**F**), and 7 d (**G**). Data are presented as mean ± SE; different letters indicate significant differences (*p* < 0.05).

The combined application of *Trichoderma* metabolites (phenol, ethyl 2, 4-dioxovalerate, and diglycolic acid) with fipronil (1 or 10 μg/g) could effectively kill termites in a concentration-dependent manner, and all treatments achieved near-100% termite mortality at 7 d (Figure 4 and Supplementary Table S2).

### 3.3. Synergistic Attract–Kill Effects of Trichoderma Metabolites on Insecticides on Coptotermes formosanus (Two-Choice Aggregation Test)

#### 3.3.1. Synergistic Attract–Kill Effects of *Trichoderma* Metabolites on Imidacloprid on *Coptotermes formosanus*

All *Trichoderma* metabolites (phenol, ethyl 2, 4-dioxovalerate, and diglycolic acid) significantly increased the percentage of termites on the treated filter paper compared to the control at 1, 2, 4, 8, and 12 h (Table 7 and Supplementary Table S3). When combined with imidacloprid (25 or 50 μg/g), these compounds also induced significantly higher percentages of termites on treated filter papers compared to the control over four time points.

**Figure 5:**
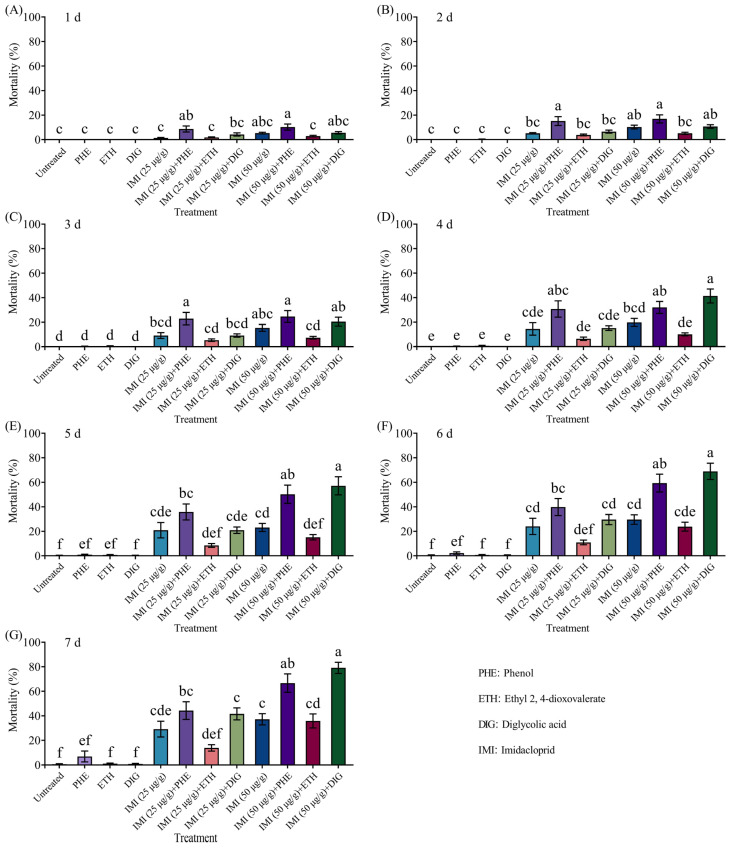
Mortality of *Coptotermes formosanus* in the aggregation choice test with the combined use of different compounds and imidacloprid at 1 d (**A**), 2 d (**B**), 3 d (**C**), 4 d (**D**), 5 d (**E**), 6 d (**F**), and 7 d (**G**). Data are presented as mean ± SE; different letters indicate significant differences (*p* < 0.05).

The combined application of *Trichoderma* metabolites (phenol, ethyl 2, 4-dioxovalerate, and diglycolic acid) with imidacloprid (25 or 50 μg/g) resulted in significant mortality in termites (Figure 5 and Supplementary Table S4). Notably, the combination of 50 μg/g of imidacloprid with two compounds (phenol and diglycolic acid) caused significantly higher mortality compared to imidacloprid alone at 4–7 d and 5–7 d, respectively.

#### 3.3.2. Synergistic Attract–Kill Effects of *Trichoderma* Metabolites on Fipronil on *Coptotermes formosanus* (Two-Choice Aggregation Test)

All *Trichoderma* metabolites (phenol, ethyl 2, 4-dioxovalerate, diglycolic acid), both alone and in combination with fipronil (1 μg/g), elicited significantly higher percentages of termites on treated filter paper compared to the control (Table 8 and Supplementary Table S5). Notably, fipronil at 10 μg/g alone caused significantly higher termite percentages on treated filter papers at three time points (1, 2, and 12 h). When co-applied with compounds, the combined treatment resulted in significantly higher termite percentages on treated filter paper relative to the control, indicating that these compounds attenuated the repellency of fipronil (10 μg/g).

**Figure 6:**
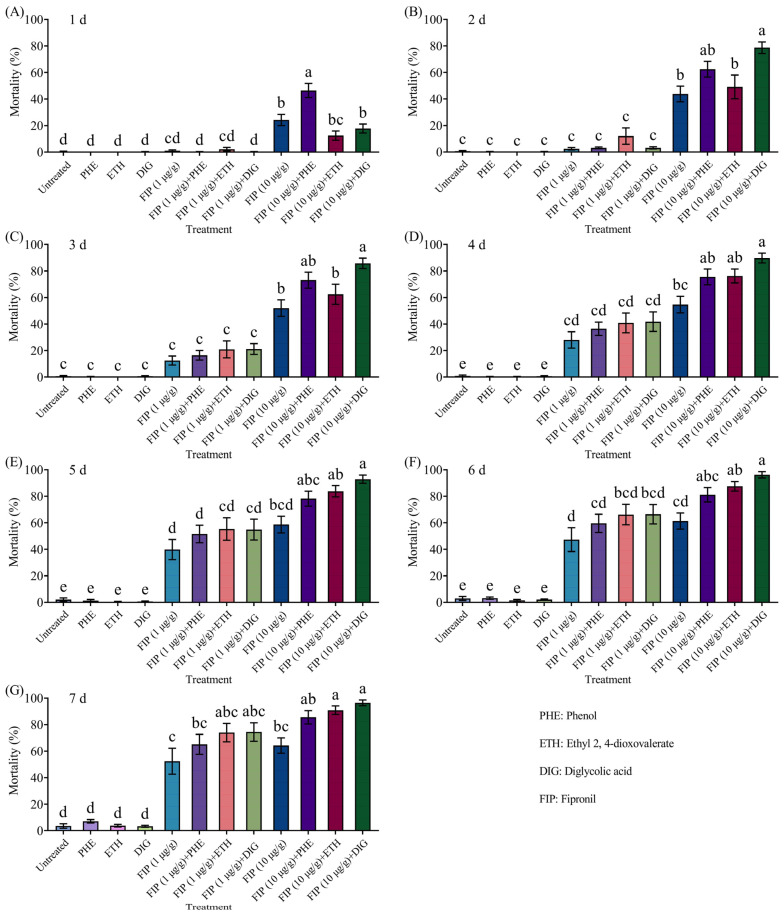
Mortality of *Coptotermes formosanus* in the aggregation choice test with the combined use of different compounds and fipronil at 1 d (**A**), 2 d (**B**), 3 d (**C**), 4 d (**D**), 5 d (**E**), 6 d (**F**), and 7 d (**G**). Data are presented as mean ± SE; different letters indicate significant differences (*p* < 0.05).

The mortality of termites increased proportionally with the fipronil concentration in a concentration-dependent manner, regardless of whether it was combined with *Trichoderma* metabolites (Figure 6 and Supplementary Table S5). Of those, when the concentration of fipronil was 10 μg/g, the mortality of termites was significantly higher than fipronil alone at 1 d, 6–7 d, and 2–7 d after being combined with phenol, ethyl 2, 4-dioxovalerate, and diglycolic acid, respectively.

## 4. Discussion

This study investigated the behavioral regulatory effects of three *Trichoderma* metabolites (phenol, ethyl 2, 4-dioxovalerate, and diglycolic acid) on *C*. *formosanus* and their synergistic interactions with insecticides. Our study showed that ethyl 2, 4-dioxovalerate and diglycolic acid showed no lethal effects, while phenol (5 × 10^−2^ to 5 μg/cm) and ethyl 2, 4-dioxovalerate (5 × 10^−1^ μg/cm) demonstrated trail-following activity. All *Trichoderma* metabolites (phenol, ethyl 2, 4-dioxovalerate, and diglycolic acid) had synergistic effects with insecticides. Specifically, phenol or diglycolic acid showed potentiation effects on imidacloprid, while metabolites (phenol or diglycolic acid)–imidacloprid, and metabolites (ethyl 2, 4-dioxovalerate or diglycolic acid)–fipronil treatments exhibited significant attract–kill effects.

Attractants have long been a central research focus in termite control. Previous studies have identified that specific chemical components, such as K_2_CO_3_, CO_2_, and 2-phenoxyethanol, exhibited attractant properties on Rhinotermitidae [17,25,26]. For instance, 2-phenoxyethanol triggered trail-following behavior at concentrations of 0.023–2.3 μg/cm and significantly stimulated tunneling behavior at 0.082% (*w*/*v*) [17,19]. Chemicals secreted by insects were shown to act as termite attractants. *Reticulitermes speratus* utilized even-numbered fatty acids (palmitic acid and trans-vaccenic acid) to mediate aggregation behavior, while *C*. *formosanus* workers secreted odd-numbered fatty ((Z)-10-heptadecenoic acid) and even-numbered fatty acids (palmitoleic acid, palmitic acid, and stearic acid) for similar functions [27,28]. Wood-decayed fungus metabolites also served as termite attractants. Matsumura et al. [29] reported that *Gloeophyllum trabeum* produced (Z,Z,E)-3,6,8-dodecatrien-1-ol, a termite trail pheromone, while Su [30] demonstrated that acetone extracts of *G. trabeum* induced directional movement in termites. Previously, authors analyzed and identified phenol, ethyl 2, 4-dioxovalerate, and diglycolic acid from *Trichoderma* metabolites. At 1000 μg/mL, these compounds significantly increased the percentage of termites on the treated filter paper compared to controls throughout experiments (24 h), indicating aggregation behavior [20]. These findings align with the two-choice aggregation test results in this study, where all three compounds elicited the aggregation behavior of termites at five time points (1, 2, 4, 8, and 12 h). Particularly, phenol and ethyl 2, 4-dioxovalerate can also induce the trail-following behavior of termites. These results demonstrate that these three compounds could attract *C*. *formosanus* in multiple ways, which could lead to enhanced efficacy in applications by directing termites to target zones through various behavioral channels.

Attractants, as insect behavior regulators, can induce target insects to exhibit behavioral responses such as aggregation, trail-following, and feeding, effectively enhancing their efficiency in locating and utilizing resources [31]. Based on this biological characteristic, current pest control strategies establish synergistic systems combining attractants and insecticides to enhance the targeting capability and persistence of insecticides, providing efficient and environmentally friendly technical solutions for integrated pest management (IPM) [9,15]. However, the combined application of attractants and insecticides must satisfy two key conditions: attractants should not interfere with the physiological activity of target insects or trigger behavioral avoidance, while also ensuring the combination acts synergistically to enhance toxicity. In this study, except for high-concentration phenol (2500 μg/g), the tested concentrations of *Trichoderma* metabolites (ethyl 2, 4-dioxovalerate and diglycolic acid) did not significantly affect termite survivorship. This indicates that these *Trichoderma* metabolites could effectively regulate termite behavior without imposing excessive burdens on non-target organisms or the environment. Critically, these metabolites did not induce a feeding preference in termites. This indicates that their action primarily targets the communication and aggregation systems of *C. formosanus*, not feeding stimulation pathways, which is distinct from the mechanism of traditional feeding-based baits. This mechanistic difference underscores their potential for safe and sustainable integration into insecticide-based control systems.

Ahmad et al. [9] reported that when attractants and insecticides were used in combination, these groups could induce behaviors such as aggregation, trail-following, feeding, and tunneling, thereby concentrating dispersed termites and prolonging their exposure to insecticides, ultimately enhancing termite mortality. For example, 2-phenoxyethanol, when used as a termite attractant in combination with insecticides, caused *C. formosanus* to remain in insecticide-treated areas for extended periods, resulting in significantly higher mortality rates compared to insecticide use alone [17,18,19]. The results of this study show that the tested compounds exhibit potentiation effects with insecticides. In no-choice insecticide synergy tests, the combination of phenol with imidacloprid (25 or 50 μg/g) resulted in mortality rates of 91% and 95% for *C. formosanus* on day 7, significantly higher than those achieved with imidacloprid alone (44% and 59%, respectively). Similarly, the combination of diglycolic acid and imidacloprid (50 μg/g) yielded an 80% mortality rate on day 7, also significantly surpassing the efficacy of imidacloprid alone. Furthermore, the combined use of attractants and insecticides could exert an attract–kill effect. In two-choice aggregation tests, all compounds (phenol, ethyl 2, 4-dioxovalerate, and diglycolic acid) combined with insecticides (imidacloprid and fipronil) consistently induced the aggregation behavior of *C. formosanus*. These *Trichoderma* metabolites were able to attenuate or eliminate the repellency of fipronil (10 μg/g) while maintaining continuous attraction of termites. The combination of phenol and imidacloprid (50 μg/g) produced a 95% mortality rate after 7 days, which is significantly higher than that of imidacloprid alone (59%). Similarly, diglycolic acid combined with imidacloprid (50 μg/g) achieved a mortality rate of 79.11%, markedly exceeding that of imidacloprid alone. When high-concentration *Trichoderma* metabolites (ethyl 2, 4-dioxovalerate and diglycolic acid) were combined with fipronil (10 μg/g), mortality rates of 91% and 96% were observed after 7 days, significantly higher than the 64% mortality rate caused by fipronil alone. Among these, the attract-and-kill effect of ethyl 2, 4-dioxovalerate combined with fipronil was consistent with the results reported in [21]. These results indicate that the combined use of *Trichoderma* metabolites and insecticides effectively prolongs the retention of *C. formosanus* in insecticide-treated zones, substantially improving control efficacy against this termite species. This mechanism, which enhances efficacy through behavioral regulation, provides a more environmentally friendly and sustainable alternative to traditional reliance on increasing pesticide concentration, as it reduces chemical usage and environmental impact. Despite the notable results achieved in this study, several limitations should be acknowledged. In future work, we plan to conduct soil-based assays to systematically evaluate the potential of combining *Trichoderma* metabolites with insecticides for field control of *C. formosanus*.

## 5. Conclusions

In summary, this study demonstrated that the three *Trichoderma* metabolites (phenol, ethyl 2, 4-dioxovalerate, and diglycolic acid) exhibited both behavioral regulatory activity and safety, making them promising candidates for developing novel termite control agents. These metabolites synergistically enhance the efficacy of insecticides, thereby improving control efficiency against *C. formosanus*. Future research should focus on elucidating their mechanisms of action, optimizing formulation designs, and advancing the practical application of fungal-derived compounds in precision-based termite management strategies.

## Figures and Tables

**Table 1:** Tests on the synergistic effect of different compounds and imidacloprid on the control of *Coptotermes formosanus*.

Treatment	Filter Paper
1	Control (only treated with acetone)
2	Phenol (2500 μg/g)
3	Ethyl 2, 4-dioxovalerate (2500 μg/g)
4	Diglycolic acid (2500 μg/g)
5	Imidacloprid (25 μg/g)
6	Imidacloprid (25 μg/g) and phenol (2500 μg/g)
7	Imidacloprid (25 μg/g) and ethyl 2, 4-dioxovalerate (2500 μg/g)
8	Imidacloprid (25 μg/g) and diglycolic acid (2500 μg/g)
9	Imidacloprid (50 μg/g)
10	Imidacloprid (50 μg/g) and phenol (2500 μg/g)
11	Imidacloprid (50 μg/g) and ethyl 2, 4-dioxovalerate (2500 μg/g)
12	Imidacloprid (50 μg/g) and diglycolic acid (2500 μg/g)

**Table 2:** Tests on the synergistic effect of different compounds and fipronil on the control of *Coptotermes formosanus*.

Treatment	Filter Paper
1	Control (only treated with acetone)
2	Phenol (2500 μg/g)
3	Ethyl 2, 4-dioxovalerate (2500 μg/g)
4	Diglycolic acid (2500 μg/g)
5	Fipronil (1 μg/g)
6	Fipronil (1 μg/g) and phenol (2500 μg/g)
7	Fipronil (1 μg/g) and ethyl 2, 4-dioxovalerate (2500 μg/g)
8	Fipronil (1 μg/g) and diglycolic acid (2500 μg/g)
9	Fipronil (10 μg/g)
10	Fipronil (10 μg/g) and phenol (2500 μg/g)
11	Fipronil (10 μg/g) and ethyl 2, 4-dioxovalerate (2500 μg/g)
12	Fipronil (10 μg/g) and diglycolic acid (2500 μg/g)

**Table 3:** Tests on the combined effect of different compounds and imidacloprid on the attract–kill effects of *Coptotermes formosanus*.

Two-Choice Aggregation Test	Treated Filter Paper	Untreated Filter Paper
1	Control (only treated with acetone)	Only treated with acetone
2	Phenol (2500 μg/g)	Only treated with acetone
3	Ethyl 2, 4-dioxovalerate (2500 μg/g)	Only treated with acetone
4	Diglycolic acid (2500 μg/g)	Only treated with acetone
5	Imidacloprid (25 μg/g)	Only treated with acetone
6	Imidacloprid (25 μg/g) and phenol (2500 μg/g)	Only treated with acetone
7	Imidacloprid (25 μg/g) and ethyl 2, 4-dioxovalerate (2500 μg/g)	Only treated with acetone
8	Imidacloprid (25 μg/g) and diglycolic acid (2500 μg/g)	Only treated with acetone
9	Imidacloprid (50 μg/g)	Only treated with acetone
10	Imidacloprid (50 μg/g) and phenol (2500 μg/g)	Only treated with acetone
11	Imidacloprid (50 μg/g) and ethyl 2, 4-dioxovalerate (2500 μg/g)	Only treated with acetone
12	Imidacloprid (50 μg/g) and diglycolic acid (2500 μg/g)	Only treated with acetone

**Table 4:** Tests on the combined effect of different compounds and fipronil on the attract–kill effects of *Coptotermes formosanus*.

Two-Choice Aggregation Test	Treated Filter Paper	Untreated Filter Paper
1	Control (only treated with acetone)	Only treated with acetone
2	Phenol (2500 μg/g)	Only treated with acetone
3	Ethyl 2, 4-dioxovalerate (2500 μg/g)	Only treated with acetone
4	Diglycolic acid (2500 μg/g)	Only treated with acetone
5	Fipronil (1 μg/g)	Only treated with acetone
6	Fipronil (1 μg/g) and phenol (2500 μg/g)	Only treated with acetone
7	Fipronil (1 μg/g) and ethyl 2, 4-dioxovalerate (2500 μg/g)	Only treated with acetone
8	Fipronil (1 μg/g) and diglycolic acid (2500 μg/g)	Only treated with acetone
9	Fipronil (10 μg/g)	Only treated with acetone
10	Fipronil (10 μg/g) and phenol (2500 μg/g)	Only treated with acetone
11	Fipronil (10 μg/g) and ethyl 2, 4-dioxovalerate (2500 μg/g)	Only treated with acetone
12	Fipronil (10 μg/g) and diglycolic acid (2500 μg/g)	Only treated with acetone

**Table 5:** Distance followed (cm; max 10 cm; *n* = 60) by *Coptotermes formosanus* in bioassays with artificial trails of different compounds.

Trichoderma Metabolite		Phenol	Ethyl 2, 4-dioxovalerate	Diglycolic Acid
Concentration (μg/cm)	5 × 10^−4^	1.3 ± 0.2 c	1.6 ± 0.2 b	1.8 ± 0.3 a
	5 × 10^−3^	2.1 ± 0.3 c	1.5 ± 0.2 b	1.4 ± 0.2 a
	5 × 10^−2^	**4.6 ± 0.5** b	1.7 ± 0.2 b	1.8 ± 0.3 a
	5 × 10^−1^	**9.5 ± 0.2** a	**3.8 ± 0.4** a	1.7 ± 0.3 a
	5	**8.7 ± 0.4** a	1.2 ± 0.3 b	2.2 ± 0.3 a
	5 × 10^1^	1.8 ± 0.5 c	0.3 ± 0.2 c	1.8 ± 0.3 a
Statistical results		*χ*^2^ = 194.99; df = 5; *p* < 0.0001	*χ*^2^ = 94.25; df = 5; *p* < 0.0001	*χ*^2^ = 4.88; df = 5; *p* = 0.4307

Note: The data in the table are the mean ± SE. Distances in bold font represent the activity thresholds which are the concentrations triggering termites to walk a mean distance of over 3 cm. Different letters in the same column indicate significant differences (*p* < 0.05).

**Table 6:** Wood consumption (mean ± SE) of *Coptotermes formosanus* in response to blocks treated with different concentrations of different compounds.

*Trichoderma* Metabolite		Phenol	Ethyl 2, 4-dioxovalerate	Diglycolic Acid
Concentration (μg/g)	0	262.09 ± 26.13 a	138.67 ± 21.08 a	168.26 ± 32.77 a
	25	353.65 ± 61.84 a	238.53 ± 35.29 a	160.83 ± 31.56 a
	250	313.67 ± 63.10 a	205.38 ± 29.54 a	166.53 ± 30.07 a
Statistical results		*F* = 0.22; df = 2, 27; *p* = 0.8074	*F* = 3.01; df = 2, 27; *p* = 0.0658	*F* = 0.01; df = 2, 27; *p* = 0.9852

Note: The data in the table are the mean ± SE. The same letter indicates in the column no significant difference.

**Table 7:** Aggregation choice of *Coptotermes formosanus* in response to the combined use of different compounds and imidacloprid.

Two-Choice Aggregation Test	Aggregation Choice of Termite at Different Time Points				
	1 h	2 h	4 h	8 h	12 h
Untreated					
Phenol (2500 μg/g)	+	+	+	+	+
Ethyl 2, 4-dioxovalerate (2500 μg/g)	+	+	+	+	+
Diglycolic acid (2500 μg/g)	+	+	+	+	+
Imidacloprid (25 μg/g)		+	+		
Imidacloprid (25 μg/g) and phenol (2500 μg/g)	+	+	+	+	+
Imidacloprid (25 μg/g) and ethyl 2, 4-dioxovalerate (2500 μg/g)	+		+	+	+
Imidacloprid (25 μg/g) and diglycolic acid (2500 μg/g)	+	+	+	+	+
Imidacloprid (50 μg/g)					
Imidacloprid (50 μg/g) and phenol (2500 μg/g)	+	+	+	+	+
Imidacloprid (50 μg/g) and ethyl 2, 4-dioxovalerate (2500 μg/g)	+	+	+	+	+
Imidacloprid (50 μg/g) and diglycolic acid (2500 μg/g)		+	+	+	+

Note: +, significantly more termites aggregated on the treated filter paper discs than untreated discs; blank entries indicate no significant difference between treated and untreated filter paper discs. Data and statistical results of termites can be found in Table S3.

**Table 8:** Aggregation choice of *Coptotermes formosanus* in response to the combined use of different compounds and fipronil.

**Two-Choice Aggregation Test**	**Aggregation Choice of Termite at Different Time Points**				
	**1 h**	**2 h**	**4 h**	**8 h**	**12 h**
Untreated					
Phenol (2500 μg/g)	+	+	+	+	+
Ethyl 2, 4-dioxovalerate (2500 μg/g)	+	+	+	+	+
Diglycolic acid (2500 μg/g)	+	+	+	+	+
Fipronil (1 μg/g)					
Fipronil (1 μg/g) and phenol (2500 μg/g)	+	+		+	+
Fipronil (1 μg/g) and ethyl 2, 4-dioxovalerate (2500 μg/g)	+	+	+	+	+
Fipronil (1 μg/g) and diglycolic acid (2500 μg/g)			+	+	+
Fipronil (10 μg/g)	−	−			−
Fipronil (10 μg/g) and phenol (2500 μg/g)	+	+	+	+	+
Fipronil (10 μg/g) and ethyl 2, 4-dioxovalerate (2500 μg/g)			+	+	+
Fipronil (10 μg/g) and diglycolic acid (2500 μg/g)		+	+	+	+

Note: +, significantly more termites aggregated on the treated filter paper discs than untreated discs; −, significantly fewer termites aggregated on the treated filter paper discs than untreated discs; blank entries indicate no significant difference between treated and untreated filter paper discs. Data and statistical results of termites can be found in Table S3.

## Data Availability

The authors confirm that the data supporting the findings of this study are available within the article [and/or] as its Supplementary Materials.

## Supplementary Materials

The following supporting information can be downloaded at https://www.mdpi.com/article/10.3390/insects16111116/s1, Figure S1: Bioassay arenas for evaluating the three-choice feeding preference tests; Table S1: Statistical results of Figure 3; Table S2: Statistical results of Figure 4; Table S3: Percentage (mean ± SE) of *Coptotermes formosanus* aggregated at different locations in the two-choice aggregation test involving with either or both *Trichoderma* metabolites and imidacloprid at 1, 2, 4, 8, and 12 h (Statistical results of Table 7); Table S4: Statistical results of Figure 5; Table S5: Percentage (mean ± SE) of *Coptotermes formosanus* aggregated at different locations in the two-choice aggregation test involving with either or both *Trichoderma* metabolites and fipronil at 1, 2, 4, 8, and 12 h (Statistical results of Table 8); Table S6: Statistical results of Figure 6.
