# Supplementary figures and images for: Attraction-Mediated Synergy: Insecticide Toxicity Against Coptotermes formosanus Enhanced by Trichoderma Metabolites
Source: Insects. 2025 Oct 31;16(11):1116. doi: 10.3390/insects16111116 (PMC12653544; doi:10.3390/insects16111116)

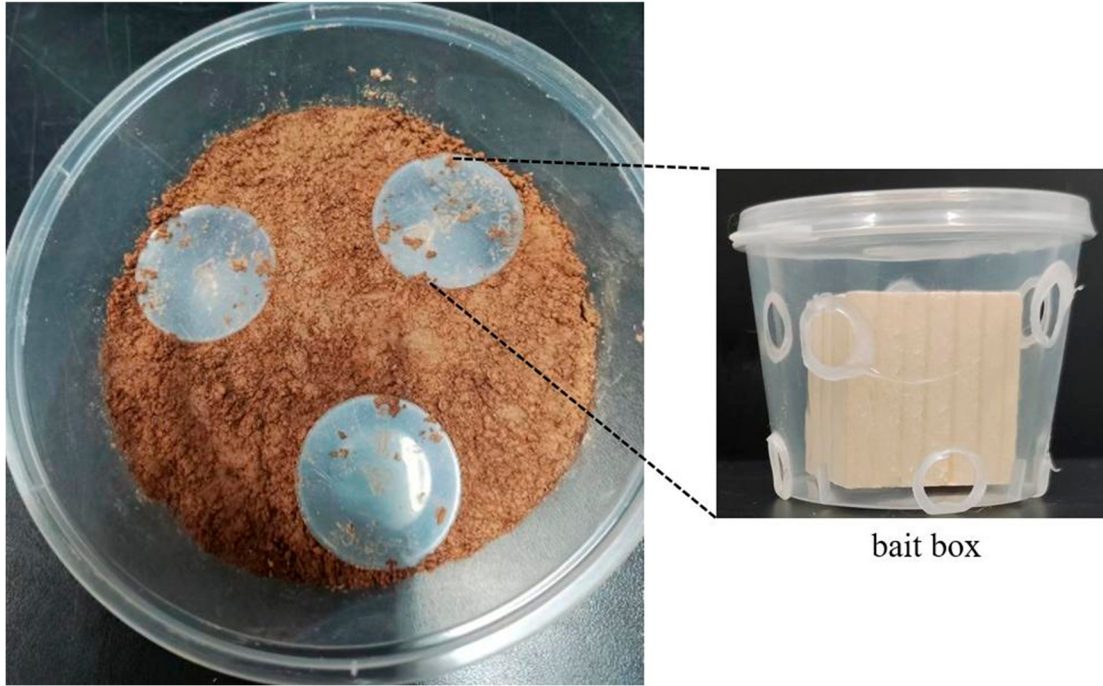

Figure S1 Bioassay arenas for evaluating the three-choice feeding preference tests

Supplement: Supplementary file 1 [file insects-16-01116-s001.zip › Figure S1.pdf]
